# Influence of socio-affective factors on quality of life in women diagnosed with fibromyalgia

**DOI:** 10.3389/fpsyg.2023.1229076

**Published:** 2023-11-08

**Authors:** Ana Raquel Ortega-Martínez, María Luisa Grande-Gascón, María José Calero-García

**Affiliations:** ^1^Department of Psychology, Universidad de Jaén, Jaén, Spain; ^2^Faculty of Health Sciences, Universidad de Jaén, Jaén, Spain

**Keywords:** fibromyalgia, quality of life, partner relationship, loneliness, socio-affective factors

## Abstract

**Introduction:**

Fibromyalgia is a disease that involves chronic pain, with high prevalence in the female population and great impact on the bio-psycho-social sphere of people affected by it. However, few studies have analyzed the possible influence of socio-affective factors on the quality of life of people who suffer from this disease.

**Objective:**

The aim of this study was to determine the relationships between the impact of this disease on the lives of people with fibromyalgia and these variables. Specifically, we analyzed the quality of partner relationship, perceived loneliness, life satisfaction, and perceived socio-family situation.

**Method:**

A descriptive-correlational cross-sectional design was used. The sample consisted of 69 women diagnosed with fibromyalgia. The participants completed different questionnaires that measured their happiness, satisfaction with life, perceived loneliness, quality of partner relationship, socio-family valuation, and the impact of fibromyalgia.

**Results:**

The quality of partner relationship, perceived loneliness and socio-family valuation seem to be good predictors of subjective happiness, life satisfaction, and the impact that fibromyalgia has on people’s lives, in the sense that the more positive the valuation of the couple relationship and of the socio-family situation, and the lower the perceived loneliness, people feel happier, more satisfied with their lives and the lower the impact that fibromyalgia has on their lives.

**Conclusion:**

The 50% of satisfaction with life can be explained from the scores obtained in perceived loneliness and the quality of partner relationship. In this sense, perceived loneliness was a good predictor of the impact of fibromyalgia on the lives of these patients.

## 1. Introduction

Fibromyalgia (FM), a disease that had not been recognized until 1992 by the World Health Organization (WHO), was defined by the American College of Rheumatology, in 2010, by the presence a of two facts: a history of generalized pain, for at least 3 months, and pressure-induced pain in a minimum of 11 out of the 18 chosen points (nine pairs) that correspond to the most sensitive areas of the organism, for low-threshold stimuli (Wolfe et al., 2010). In 2019, this diagnostic criterion was modified and replaced by the presence of pain in six of the nine proposed body areas: head, left arm, right arm, right lower limb, left lower limb, thorax, abdomen, upper and lower back (Arnold et al., 2020). The prevalence of FM is between 2 and 5% of the population and it is more common in women, with a ratio of 9:1(Córdoba-Torrecilla et al., 2016).

Pain and fatigue are two of the most common symptoms and some studies have found a significant percentage of people presenting symptoms up to 10 years before diagnosed with this disease (Mur-Martí et al., 2016). The intensity of the pain has a negative influence on the psychological factors, such as coping with pain and catastrophizing, and social factors, such as problems in the partner relationship or in the work and/or family scope (Segura-Jiménez et al., 2016). Furthermore, this pathology is associated with an increase of dependency, a decrease of work activity, an increase of work absenteeism, increases of family problems, and the need for a larger number of social networks.

The treatment of this syndrome is complex. Pharmacological treatments act on one or several of the symptoms, but do not globally improve the disease, so patients resort to non-pharmacological treatments, such as health education, physical exercise, homeopathy and manual therapies, as well as Multicomponent Cognitive Behavioral Therapy, which addresses symptoms such as pain, mood, self-control, daily functioning, physical state, fatigue and sleep (Hooten et al., 2007; Fitzcharles et al., 2014; Andrade et al., 2019; Govillard et al., 2022). The positive results obtained in their treatment with comprehensive or multicomponent programs (i.e., not only pharmacological), coupled with the fact that healthy social interactions and support seeking have been observed to improve their physical and mental health (Restrepo et al., 2022) indicates the importance of knowing the family and social aspects that influence the perception of this disease and that, therefore, must be taken into account for its management (Galvez-Sánchez et al., 2019; Seto et al., 2019).

Different research has shown that the onset of the disease produces significant changes in the interpersonal relationships of affected individuals (Del Pozo et al., 2015; Biccheri et al., 2016; Armentor, 2017; Ataoğlu et al., 2017; Jiménez et al., 2017; Muller et al., 2017) among which the relationship with the partner takes on special importance. The problems that arise in couples due to the repercussions of the disease favor the appearance of conflicts that can have an impact on the life satisfaction and happiness of people affected by FM. Particularly in people with FM, both chronic and transient loneliness have been found to be associated with more negative social interactions and a greater experience of pain (Wolf and Davis, 2014). Therefore, objective or perceived loneliness may be an important factor to consider in this syndrome.

On the other hand, the social perception of this disease also affects women with fibromyalgia. One of the main triggers of psychological symptoms is fear and frustration due to the rejection generated by misunderstanding of the topic. That is why there is a tendency toward isolation and an increase in perceived loneliness, as a result of the relationship with doctors and the disbelief on the part of some of them in the existence of the disease (Vasquez, 2017).

In addition, a large number of studies have related pain to the quality life (QL) of people with FM, with the aim of improving the QL of these patients by proposing different interventions to reduced pain (Bernard et al., 2000; Kaplan et al., 2000; Burckhardt et al., 2001; Verbunt et al., 2008). However, a systematic review with meta-analysis (Mascarenhas et al., 2021) suggests that most of the available therapies can improve QL, reducing pain in the short and medium term, but these results are not associated with clinically important improvements for patients. Pain in FM has also been compared to that in other rheumatic diseases (Bucourt et al., 2021), concluding that, in the case of FM, the disease has a greater impact on daily life; patients had more difficulties in adapting to the disease and, in general, used poorer coping strategies to deal with pain (Beyazal et al., 2018; Restrepo et al., 2022). One variable associated with QL is life satisfaction, as it is part of the cognitive component of subjective well-being, considered as an important health indicator. Thus, low life satisfaction may be related to the development or worsening of chronic diseases (Diener and Chang, 2011; Boonstra et al., 2013). In fact, it has been found that in patients with FM, higher levels of life satisfaction are associated with better adaptation to FM (Luque-Reca et al., 2021, 2022).

Thus, according to the literature, different variables associated with interpersonal relationships and the degree of life satisfaction may be related to the severity of FM, as well as to the impact of this disease in different areas of the lives of those affected by it. However, the possible relationships between these types of variables, as well as the degree to which they can predict the impact of the disease on the lives of those who suffer from it, and therefore their QL, has been poorly studied. Therefore, the aim of this study was to determine the relationships between the impact of this disease on the lives of people with FM and these variables. Specifically, we analyzed the quality of partner relationship, perceived loneliness, life satisfaction, and perceived socio-family situation.

## 2. Materials and methods

### 2.1. Design and procedure

This was a descriptive-correlational study. The variables considered were obtained through the instruments described in the next subsection.

Once the pertinent authorizations were obtained from the Ethics Committee of Universidad de Jaén (Spain) with the code OCT.21/5.PRY. The study objectives and procedure were explained to the potential participants, indicating that their data would be recorded and analyzed anonymously and that they could drop out of the research at any time, if they wished to. Finally, they were asked to sign their informed consent.

A researcher of the research team went to the office of the association of people with FM at Jaen (Spain), where she agreed with the president of the association upon the research procedure to be followed in this study. Booklets with the instruments and instructions were prepared, designing them to be self-administered by the participants, which prevented unnecessary trips and allowed the latter to choose the time to complete the scales. Previously, the participants were given written information about the project, and they were asked to sign an informed consent form, which they were requested to attach to the booklet with the answers. The booklets were gathered in the office of the association at Jaen. In regard with the inclusion criteria, the study included people who had been diagnosed with FM at least 2 years before the beginning of the study; informed consent was also required for participation. The data obtained between November 2021 and January 2022.

### 2.2. Instruments

The instruments used to carry out the study were:

*Data gathering sheet* about the sociodemographic (sex, age, marital status, and education level of the participants) and clinical variables (medication, other pathologies, body mass index, etc.), which was completed by the research team.

*Life Satisfaction Scale* (LSS; Diener et al., 1985; Spanish version by Vázquez et al., 2013). This scale assesses the participants’ general level of satisfaction with their lives. The aim of this instrument is to evaluate the cognitive aspects of well-being. It presents five items, which are answered according to the degree of agreement with a 7-point scale. The minimum score is 5 points (very dissatisfied) and the maximum score is 35 points (totally satisfied). The psychometric properties of the LSS have been widely established in the Spanish population, showing an internal consistency of 0.87 in this study.

*Subjective Happiness Scale* (Lyubomirsky and Lepper, 1999; Spanish version by Extremera et al., 2009). This is a Likert scale of four items that measure global subjective happiness using statements through which the subject assesses him/herself or compares him/herself with other people in his/her environment. In this study, this scale showed an internal consistency coefficient (Cronbach’s α) of 0.88.

*Quality of partner relationship questionnaire* (Gottman and Silver, 2001). This questionnaire measures the perception of the quality of the relationship by one of the members of the couple. It consists of 20 dichotomous response statements (Yes/No). In the present study, this instrument presented a Cronbach’s alpha of 0.84.

*Perceived Loneliness Scale* (ESTE II, Rubio et al., 2010). Composed of 15 items with three response options (always, sometimes, and never), this scale is divided in three factors: perceived social support, the use that the respondent makes of information technologies, and social participation. The total score of the scale ranges between 0 and 30 points, and it is obtained through the sum of the score of each of the items. In this study, an internal consistency of 0.65 was obtained.

*Gijón Socio-family Valuation Scale* (Cabrera-González et al., 1999). This scale gathers five valuation areas: family situation, economic situation, housing, social networks, and support received from social networks. In this study, a Cronbach’s alpha of 0.413 was obtained.

*Fibromyalgia Impact Questionnaire* (FIQ; Burckhardt et al., 1991, Spanish version of Monterde et al., 2004). FIQ evaluates the impact of FM on physical capacity, the possibility of performing the usual activities of daily living, and, if employed or self-employed, the extent to which FM has affected the respondent’s economic activity, as well as items related to the symptoms of FM (pain, fatigue, feeling of tiredness, and stiffness) and to the emotional state of the respondent (anxiety and depression), given that the impact on these issues, such as physical capacity and others, is responsible for the impact on QL. Regarding the reliability in our sample, a Cronbach’s alpha of 0.771 was obtained.

### 2.3. Statistical analysis

A descriptive analysis of the sociodemographic variables considered (frequencies, means and standard deviations) was performed. Correlation coefficients were calculated to determine the possible relationships between the variables considered using Hmisc library (Harrell, 2021). In addition, the data were analyzed using the general linear model (library stats; R Core Team, 2021), stepwise regression analyses were performed to find out to what extent the quality of the couple relationship, perceived loneliness, and socio-family valuation can be predictors of life satisfaction, subjective happiness and impact of FM happiness. Finally, analyses of variance were carried out in order to determine the possible existence of differences in the impact of FM as a function of other variables. All statistical tests were conducted using R project for statistical computing and statistical decisions were made at a significance level of 0.05.

## 3. Results

The sample was constituted by 69 women affected by FM aged between 29 and 70 years (*M* = 56 and *SD* = 8.72), of whom 71% (*n* = 49) had a partner. Regarding their marital status, 10.1% (*n* = 7) were single, 63.8% (*n* = 44) were married, 10.1% (*n* = 7) were widowed, and 13% (*n* = 9) were divorced. With respect to their education level, 7.2% (*n* = 5) had no studies, 43.5% (*n* = 30) had completed primary education, 11.6% (*n* = 8) had completed high school education, 24.6% (*n* = 17) had completed vocational training, and 11.6% (*n* = 8) had completed higher education.

According to the sociodemographic information gathered, 84.1% of the participants (*n* = 58) lived in an urban area, whereas the rest of the participants lived in a rural area. Regarding housing, 81.2% (*n* = 56) lived in their own homes, 15.9% (*n* = 11) lived with their parents, and the rest of the participants did not specify the type of housing. With respect to coexistence, 49.3% (*n* = 34) of the participants lived with a partner, 34.8% (*n* = 24) lived with their children, 8.7% (*n* = 6) lived alone, and 7.2% (*n* = 5) stated other forms of coexistence. In regard with physical activity, 2.9% (*n* = 2) of the participants did not perform any type of activity, 7.2% (*n* = 5) only carried out self-care activities, 11.6% (*n* = 8) carried out activities of daily living, 43.5% (*n* = 30) performed light exercise, 31.9% (*n* = 22) practiced moderate exercise, and 1.4% (*n* = 1) carried out intense exercise. Table 1 presents the descriptive statistics for the different measures used in this study.

**Table 1:** Mean and standard deviation of the variables considered in the study.

	Mean	SD	Min.	Max.
Num. of drugs/day	6,29	3,47	0	19
Activity days/week	3,78	1,76	0	7
BMI	32,27	11,85	14,40	63,36
Quality of partner relationship	14,93	3,94	3	20
Subjective happiness	4,32	1,30	1	6,25
Life satisfaction	15,19	5,54	5	25
Socio-family valuation	9,22	2,87	5	16
Impact of fibromyalgia	67,65	16,72	0	93,44
Perceived loneliness	11,16	4,16	2	21

We analyzed the relationship between the sociodemographic variables and the rest of the variables, obtaining no statistically significant relationships. Table 2 shows the values of the correlation coefficient, and their statistical significance, between different variables related to QL: n° of drug/day, activity days/week, BMI, Quality of partner relationship, Subjective happiness, Life satisfaction, socio-family valuation, Impact of FM and perceived loneliness.

**Table 2:** Pearson correlation coefficients between the variables considered.

	1	2	3	4	5	6	7	8
Num. of drugs/day (1)	–							
Activity days/week (2)	−0.13	–						
BMI (3)	−0.23	−0.10	–					
Quality of partner relationship (4)	−0.29*	0.19	−0.07	–				
Subjective happiness (5)	−0.04	0.06	−0.02	0.44**	–			
Life satisfaction (6)	−0.13	0.24	−0.24	0.50**	0.68**	–		
Socio-family valuation (7)	0.04	−06	0.29*	−0.37**	−0.44**	−0.45**	–	
Impact of fibromyalgia (8)	0.17	−0.37**	0.09	−0.28*	−0.12	−0.37**	0.055	–
Perceived loneliness (9)	0.30*	−0.21	0.15	−0.37**	−0.38**	−0.61**	0.32**	0.39**

*p < 0.05. **p < 0.01.

Regression analyses were carried out. Firstly, the quality of partner relationship was used as predictor variable. The results showed that this variable, on its own, could predict 17.8% of subjective happiness (*R^2^*adjusted = 0.178; *β* = 0.44 *p* < 0.00, CI95% = [0.0.61, 0.22]), 23.4% of life satisfaction (*R^2^*adjusted = 0.234; *β* = 0.49 *p* < 0.001, CI95% = [0.38, 1.06]), 5.8% of the impact of FM (*R^2^*adjusted = 0.058; *β* = −0.27 *p* < 0.05, CI95% = [−2.32, −0.013]), and 11.9% of both socio-family valuation and perceived loneliness (*R^2^*adjusted = 0.119; *β* = −0.37 *p* < 0.01, CI95% = [−0.44, −0.08]).

By using social loneliness as predictor variable, the results of the analysis show that this variable can predict 7.3% of the number of drugs that are consumed daily (*R^2^*adjusted = 0.073; *β* = 0.29 *p* < 0.01, CI95% = [0.05, 0.45]), 11.9% of the scores in partner relationship (*R^2^*adjusted = 0.119; *β* = −0.37 *p* < 0.01, CI95% = [−0.60, −0.11]), 13.4% of subjective happiness (*R^2^*adjusted = 0.134; *β* = −0.38 *p* < 0.001, CI95% = [−0.19, −0.05]), 35.4% of life satisfaction (*R^2^*adjusted = 0.354; *β* = −0.60 *p* < 0.001, CI95% = [−1.06, −0.54]), 8.8% of socio-family valuation (*R^2^*adjusted = 0.088; *β* = 0.32 *p* < 0.01, CI95% = [0.06, 0.38]) and 13.6% of the impact of FM (*R^2^*adjusted = 0.136; *β* = 0.38 *p* < 0.001, CI95% = [0.61, 2.46]).

In the case of socio-family valuation, this variable could predict 6.30% of the body mass index (*R^2^*adjusted = 0.063; *β* = 0.29 *p* < 0.05, CI95% = [0.03, 2.41]), 11.9% of the partner relationship (*R^2^*adjusted = 0.119; *β* = −0.37 *p* < 0.01, CI95% = [−0.88, −0.16]), 18.1% of the scores in subjective happiness (*R^2^*adjusted = 0.181; *β* = −0.44 *p* < 0.001, CI95% = [−0.30, −0.10]), 19.1% of life satisfaction (*R^2^*adjusted = 0.191; *β* = −0.45 *p* < 0.001, CI95% = [−1.29, −0.45]) and 8.8% of perceived loneliness (*R^2^*adjusted = 0.088; *β* = 0.32 *p* < 0.01, CI95% = [0.12, 0.79]).

Since the three factors separately seemed to be good predictors of some of the variables related to QL, such as subjective happiness, life satisfaction and the impact of FM, we considered the possibility of developing a predictive model with these three variables. To this end, a regression analysis was performed for each of the variables that could be predicted from partner relationship, socio-family valuation and perceived loneliness (Table 3).

**Table 3:** Stepwise regression analysis for subjective happiness and life satisfaction.

Criterion variable	Models variables	B	95%CI	β	*T*	*P*	*R^2^_adj_*
Subjective happiness	(constant) Partner relationship Perceived loneliness	3.92 0.10 −0.09	[2.12, 5.72] [0.02, 0.18] [−0.16, −0.01]	0.32 −0.30	34.36 2.48 −2.34	0.000 0.016 0.023	0.243
Life Satisfaction	(constant) Perceived loneliness Partner relationship	18.18 −0.78 0.42	[11.65, 24.72] [−1.07, −0.50] [0.12, 0.72]	−0.56 0.29	5.58 −5.47 2.85	0.000 0.000 0.006	0.501
Impact of FM	(constant) Perceived loneliness	50.62 1.54	[39.67, 61.56] [0.61, 2.46]	0.38	9.24 3.32	0.000 0.001	0.136

In the case of subjective happiness, the best model was that which considered the quality of partner relationship and perceived loneliness as predictors of subjective happiness, discarding socio-family valuation. By introducing these two variables in the model, 24.3% of subjective happiness can be predicted. A similar model was obtained in the case of life satisfaction. As can be observed in Table 3, partner relationship and perceived loneliness constitute the model to predict 50.1% of life satisfaction. Lastly, the same analysis was performed using perceived loneliness and partner relationship as predictor variables of the impact of FM. The results of this analysis showed that, when these three variables are considered jointly, perceived loneliness is the only significant predictor, explaining 13.8% of the variance of this variable.

In order to determine the existence of differences in the impact of FM as a function of some sociodemographic variables, such as marital status and type of housing, single-factor variance analyses were performed, using the scores of FIQ as dependent variable. No statistically significant differences were found as a function of any of the factors considered.

## 4. Discussion

The main objective of this study was to determine the possible relationships between the impact of FM on different areas of the lives of people with this disease and the quality of the couple’s relationship, perceived loneliness, life satisfaction and the socio-family situation. In addition, the physical activity performed, the number of drugs consumed and the body mass index were taken into account.

In this study, it was found that the participants consumed an average of 6.29 drugs daily, with a maximum value of 19 different drugs consumed throughout the day as usual medication. These results are in line with those reported in other studies (Lizama-Lefno and Rojas-Contreras, 2019; Molina-Mendoza et al., 2022), which shows that women with FM consume at least one drug, and some of them even more than three drugs simultaneously (Bosch-Romero et al., 2002), thus polypharmacy is common. This finding poses high rates of healthcare resource consumption, which could be due to dissatisfaction with the treatment received, since the expected results are not obtained (Vasquez, 2017) since the medication for FM not being effective, because the pathophysiology is unknown (Hulens et al., 2018).

Regarding body mass index, the results found show a surprising fact, since the participants performed some type of physical activity, with an average of 3.78 days per week, although their average body mass index was 32.27. According to the classification of the WHO for this index, this implies that a high percentage of the sample presented overweight or obesity. Few studies explore the body mass index of people affected by FM, despite being an indicator of the physical state (Vargas-Sáenz, 2022). Nevertheless, some studies relate overweight to the sitting time caused by the disease itself (Roca et al., 2021) and in other works it has been found that obesity is a risk factor for FM (Mora-Fernández, 2022).

In addition, our results show that the participants perceived themselves to be less happy than the general population, and they are less satisfied with their lives, which is in line with the results of previous studies (Fitzcharles et al., 2014; Braun et al., 2020). In the case of happiness, it was observed that the mean score was significantly lower than that obtained for the adult population, and, in the case of life satisfaction, the average score obtained is within the range of values that indicate slight dissatisfaction in various areas of people’s lives.

It is worth highlighting the great impact of FM on the lives of the participants, with mean scores of 67.65, which indicate severe affectation above the means of previous studies, who reported a mean score of 50 points (moderate affectation; Bosch-Romero et al., 2002). However, the perceived loneliness obtained in the mentioned study was lower than that obtained in the present study, and socio-family valuation was good, presenting no social risk, which is considered to be one of the main triggers of the development of the symptoms of FM (Braun et al., 2020). Thus, it was found that the presence of support network reduces the experience of pain, and thus negative emotions and the intensity of the symptoms of depression or anxiety (Freitas et al., 2017).

We observed that the quality of partner relationship can be a determinant in many of the measures that are clearly related to QL. FM causes a bad perception toward the health state, and it negatively affects the family and social environment of the person who suffers from it, including the partner relationship (Ubago-Linares et al., 2005). The results obtained in this study allow asserting that, in general, the participants had a partner relationship that was very likely to remain stable in time. Nevertheless, it is worth mentioning that a high percentage did not represent the first partner, since they described themselves as divorced or widowed. Loss of relationship may be related to sexual dysfunction in women with FM (Yilmaz et al., 2012), which is not reflected in our study.

The obtained results in terms of relationships between the variables considered in this study show that, the greater the affectation of the disease, the shorter the activity time (measured in days per week), and the lower the satisfaction with life. Likewise, the people who are less satisfied with their lives show a greater feeling of loneliness and a worse socio-family valuation. The correlation pattern of subjective happiness was similar to that of life satisfaction, presenting a strong positive correlation with the latter, and a negative correlation with both perceived loneliness and socio-family valuation. These results are in line with studies that show the relationship between the lack of understanding of the disease, the loss of social contact, and social pain, with great impact on the life satisfaction of these women (Bernard et al., 2000; Peterson, 2007). This disease associated with chronic pain is an important health problem that causes high social and personal costs and is associated with high levels of emotional distress, which leads to a clear deterioration in the different facets of well-being (Breivik et al., 2013).

Among these results, it is worth highlighting the relationships found for different variables with the quality of partner relationship, perceived loneliness, and socio-family valuation. Thus, the better the perception toward the quality of partner relationship, the better the score in subjective happiness, life satisfaction, socio-family valuation, and perceived loneliness, and the lower the number of drugs consumed and the impact of the disease on the life of the person affected by it. Therefore, it seems that the quality of partner relationship can be a determinant in many of the variables measured that are clearly related to the QL, which is in line with other studies (Pagán Vélez, 2020).

Similar results were obtained for perceived loneliness. The results show that there is a strong relationship between this variable and those related to the QL. In this sense, the lonelier the person perceives him/herself, the larger the number of drugs he/she consumes and the greater the impact of FM on his/her life, and the lower his/her happiness and life satisfaction. Similarly, the greater the perceived loneliness, the worse the perception toward the partner and socio-family relationships. Likewise, the participants with the worst socio-family assessment and greater perceived loneliness, present a higher body mass index and have worse scores in quality of relationship, subjective happiness and satisfaction with life.

Various investigations (Cardona-Arias et al., 2014; Roca et al., 2021) reported that FM has a negative impact on the physical component of QL, and that the greatest deterioration was observed in sedentary people, although in this study social factors were less affected compared to our study. Our results are in line with those of other investigations (Türkoğlu and Selvi, 2020) on the use of coping strategies and multicomponent therapies to improve QL in people with FM, indicating that they could reduce drug therapy and therefore dependence on polypharmacy.

This study shows that factors related to the perception of women with FM toward their partner relationship and loneliness are clearly related to positive affective factors. Thus, the results of this work demonstrate that these two variables are good predictors of both subjective happiness and life satisfaction. It is worth highlighting that 50% of life satisfaction can be explained from the scores obtained for perceived loneliness and the quality of partner relationship. Other works on QL, carried out with the general population, have highlighted the importance of the role of other factors in explaining life satisfaction. Thus, Lachmann et al. (2017) consider that Neuroticism and Extraversion are often the strongest predictors of life satisfaction and Malvaso and Kang (2022) proposed an integrate model with demographic, personality, and areas of life satisfaction as predictors explained of overall life satisfaction. It would be very interesting to incorporate these proposals into the study of the QL of FM patients and compare the results with those obtained in these studies.

The present study has some limitations. First, the participants in the study belong to the same association of patients with FM and the sample size is not large. Nevertheless, the sample was similar to that of other studies (Bosch-Romero et al., 2002; Zhang, 2020), although there are other studies with smaller samples (Sixto-Sueiras et al., 2019). Secondly, a cross-sectional-correlational design was used, which precludes making inferences about the causality of the relationship between the factors included. Third, the data were collected with self-reported measures, which poses a tangible risk of bias (Wold et al., 2013) and one of the scales used did not show a very high reliability index, although this has been similar to that obtained in the original study of the scale (Cabrera-González et al., 1999). Thirdly, factors that may help explain part of the results obtained, such as the different areas of life satisfaction and personality factors, have not been considered in this study (Malvaso and Kang, 2022). Fourth, we did not take into account the type of treatment that participants may have been receiving (physical exercise, medication, therapy), which may have influenced their responses.

Despite the limitations mentioned in the previous paragraph, the results of the present study show the importance of considering that the quality of the couple relationship and perceived loneliness contribute to the evaluation that people affected by FM make of their happiness (*R^2^* = 0.243) and their satisfaction with life (*R^2^* = 0.501). These findings have important theoretical implications as they contribute to the advancement of knowledge of those variables involved in the subjective assessment that people make of their life satisfaction, as well as their happiness. In addition, this study highlights the fact of considering various factors related to the socio-affective area in the treatment of the disease.

In conclusion, the results obtained pave the way for future research that should explore which factors, including sociodemographic factors like health, job, household, family, age, gender, psychological characteristics, lifestyle, leisure activity involvement, and leisure enjoyment are involved in the QL of patients with FM in order to incorporate them into an integrative model that allows better treatment of the disease.

## Data availability statement

The original contributions presented in the study are included in the article/supplementary material, further inquiries can be directed to the corresponding author.

## Ethics statement

The studies involving humans were approved by Comite de Ética de la Universidad de Jaén (OCT.21/5.PRY). The studies were conducted in accordance with the local legislation and institutional requirements. The participants provided their written informed consent to participate in this study.

## Author contributions

All authors contributed to conception, design of the study, organization the database. AO-M and MC-G performed the statistical analysis and have written the first draft of the manuscript and sections of the manuscript. All authors contributed to the article and approved the submitted version.
